# The effectiveness and acceptability of digital health interventions as tools to promote physical activity in primary care: an update scoping review

**DOI:** 10.1017/S1463423625100339

**Published:** 2025-08-15

**Authors:** Callum Leese, Kirstin Abraham, Chris van de Konijnenburg, Hussain Al-Zubaidi

**Affiliations:** 1 University of Dundee Division of Medical Sciences: University of Dundee School, UK; 2 NHS Tayside, UK; 3 NHS Grampian, UK; 4 Royal College of General Practitioners, UK

**Keywords:** Digital health, Primary Care, PA

## Abstract

**Background:**

Physical activity (PA) promotion in primary healthcare is an effective way of addressing population-based physical inactivity. Advancements in technology could help overcome barriers to promoting PA. This scoping review aims to provide an overview of technology (digital health) for PA promotion in primary healthcare, including effectiveness and acceptability, from research published between January 2020 and December 2023.

**Methods:**

A scoping review was conducted across five databases (Cochrane library, Embase, MEDLINE, PubMed and WebofScience). Search terms focused on three components: PA counselling, technology and primary healthcare. Articles from 01/01/2020 to 05/12/2023 were included. Paediatric populations and populations with diseases requiring specialist care were excluded.

**Results:**

Of 2717 studies identified during database searches, twenty-nine were included in the review. Mobile-phone applications were the preferred method of implementation (*n* = 12, 52%), with most interventions aiding in assessment of PA levels (*n* = 16, 70%) and/or assisting in addressing it (via education, monitoring or support) (*n* = 22, 96%). Findings revealed mixed evidence on the effectiveness of digital health interventions in increasing PA but reported widespread acceptability of digital health interventions. Qualitative studies revealed three main themes desired by stakeholders: (1) ease of use, (2) complements pre-existing primary healthcare provision and (3) patient-centred.

**Conclusion:**

Future research should focus on developing standardised approaches for assessing digital health interventions, exploring the impact on prescribing behaviours and addressing the desired features highlighted by stakeholders. Integration of technology in healthcare, including PA promotion, holds promise for enhancing access and facilitating widespread implementation.

## Key Take Home

Digital Health interventions for PA promotion in primary healthcare are acceptable, but their effectiveness varies. Qualitative studies show what stakeholders desire from digital health interventions: easy to use, complements pre-existing primary care provision, and is patient-centred.

## Introduction

Regular physical activity (PA) results in wide ranging physical and mental health benefits (Hardman and Stensel, 2009). Despite this, one third of adults in the European Union fail to meet the PA guidelines as defined by the World Health Organisation (WHO) (Bull *et al*., 2020), with almost half (45%) reporting they never exercise or play sport (OECD and WHO, 2023). This is important, as physical inactivity has a large detrimental impact on healthcare services, which are already stretched by the increasing burden of non-communicable diseases (Bull *et al*., 2022).

Primary healthcare professionals have wider exposure to the whole population than any other health professional – regularly seeing those in need of PA advice and viewed by the public as a trusted source of information (Lion *et al*., 2019; McNally, 2015). Given this level of exposure, it is not surprising that PA promotion delivered via primary healthcare has been shown to be effective at increasing PA in patients (Kettle *et al*., 2022) and is cost-effective (Campbell *et al*., 2015). PA promotion in healthcare settings can take a number of different formats, but in general refers to PA counselling, PA on prescription or exercise referral (Orrow *et al*., 2012). Acknowledging the cost-effectiveness of PA promotion in primary healthcare, the WHO for Europe highlighted PA counselling in primary healthcare as one of its ‘best buys’ in an economic analysis of cost per disability-adjusted life years averted (WHO, 2017). Despite primary healthcare being a key point of influence for PA behaviours, evidence shows poor implementation of PA promotion by general practitioners (GPs) (Barnes and Schoenborn, 2012; Chatterjee *et al*., 2017).

The development of technology has the potential to address many of the barriers to promotion of PA in primary healthcare (Kennedy and Hales, 2018). A scoping review by Wattanapisit and colleagues (Wattanapisit *et al*., 2020) explored the usability and utility of technology for delivering PA promotion in primary healthcare. It found mixed findings on usability and utility of technology in assisting with PA promotion, with the major barriers to use included complexity and technical issues. Since the publication of this scoping review, the world has experienced a global pandemic (COVID-19), necessitating accelerated technological advancement in health sectors. Consequently, this study aimed to update the scoping review performed in 2020, providing an overview of digital health interventions tailored for PA counselling in primary healthcare and investigated their acceptability and effectiveness. The secondary aim was to categorise the method of action of the digital health interventions according to a recognised behaviour change model.

## Methods

This scoping review was registered in the Open Science Framework (DOI 10.17605/OSF.IO/R26QB). The scoping review was conducted according to the recommendations of the Preferred Reporting Items for Systematic Reviews and Meta-Analyses (PRISMA) extension for Scoping Reviews (Tricco *et al*., 2018) and was modelled on a previous review (Wattanapisit *et al*., 2020).

### Search strategies

A systematic search strategy was performed across five databases: Cochrane Library, Embase, MEDLINE, PubMed and Web of Science (from 01/01/2020 until 05/12/2023). The search terms consisted of three components: PA counselling, technology and primary healthcare. This is outlined in Table 1.

**Table 1.** Search terms

Search Component	Search Term
Physical activity counselling	[“physical activity” OR “physical activities” OR “physical active” OR “physical exercise” OR exercise] AND [counselling OR counseling OR prescribing OR prescription OR advise OR advice OR educat*]
Technology	eHealth OR “electronic health” OR computer OR computer-based OR computer-based OR device OR phone OR smartphone OR “mobile phone” OR “cell phone” OR mHealth OR “mobile health” OR app OR application OR web OR website OR web-based OR digital OR “digital health”
Primary healthcare	“primary care” OR “primary health care” OR “primary healthcare” OR “family practice” OR “family medicine” OR “general practice” OR “general practitioner” OR GP

### Study eligibility

Studies were included if they were: (1) original published peer-review quantitative or qualitative articles conducted in primary healthcare settings, (2) technical or governmental documents (3) published in English and (4) published between 1^st^ January 2020 and 5^th^ December 2023. Studies were excluded if: (1) they consisted of review articles, opinion excerpts, protocols, conference abstracts, (2) full-text was not accessible after all avenues exhausted, (3) participants included paediatric populations or (4) participants had specific diseases requiring specialist care (e.g. cancers, chronic obstructive pulmonary disease).

For the purpose of this review, digital health was defined in accordance with the *WHO Global Strategy on Digital Health (WHO, 2021)* and includes mobile-phone applications, websites and wearable technology.

### Selection of evidence sources

After duplicate removal, the titles, abstract and full texts were screened by a single reviewer, with 10% selected for a second review, to check for discrepancies in agreement. Any discrepancies were resolved on discussion between authors. The Rayyan software was used to manage the studies found, with the final included studies exported to Endnote citation manager version 20. One author performed data extraction using an extraction template file.

### Quality appraisal

Study quality was assessed using the Mixed Methods Appraisal Tool (MMAT) Version 2018, accounting for the wide range of study types included (Hong *et al*., 2018). The MMAT tool is designed for mixed method research, but is also suitable for use within qualitative research, quantitative descriptive studies and both randomised and non-randomised studies. The MMAT uses five quality criteria that differ according to the type of study, with the possible outcome from whether each criterion being met ‘yes’, ‘no’ and ‘can’t tell’. The quality of included studies was assessed using the appropriate category for the study type. Each article was appraised by a single author and scored (using the 0–5 scoring system). A score out of 5 was provided for each study (see Table 2, summary of included studies) as a guide to study quality.

**Table 2.** summary of the included studies

Authors, year, country	MMAT score	Study design	Participant and setting	Counselling domain	Methods	eHealth intervention stage (as per 5As)	Outcomes
Agarwal *et al*., 2020, Canada	4/5	Pilot RCT	530 adults recruited from 1 primary-care centre	PA	4 month intervention comparing a web-based toolkit assessing PA levels and customized providing tailored resources and prescription versus standard care.	Ask, Advising, Assisting	MET-minutes per week at exit was no significant across the intervention (cluster 1 1412, cluster 2 732, cluster 3 292 and cluster 4 3391). Similarly, no change in self efficacy (*p* = 0.38).
Agher *et al*., 2022, France	4/5	Mixed-methods feasibility study	52 adults recruited online	PA, diet, alcohol and smoking	Mixed methods study involving an education mobile app toolkit to assess risk factors and then recommend personalized interventions. No control group.	Ask, Advising, Assisting	uMARs score 3.7 for engagement : majority found app interesting, appropriate and entertaining. However lack of customizability. uMARs 4.4 for functionality: >80% appreciated design and performance of the app. uMARS3.7 for aesthetics: >3/4 approved of layout, although graphics felt to not be appeal by >50% uMARS 4.2 for infromation: >75% approved on information content Perceived impact: 80% felt app made them aware of need to address health behaviour
Bliudzius *et al*., 2022, Lithuania	4/5	Prospective cohort study	30 adults with pre-diabetes recruited via primary care	PA	6-month intervention assessing impact of wearable tracker with associated mobile-app to assess daily physical activity levels. No CG.	Assisting	All 30 participants completed the study. Outcome measures included PA, HbA1c and triglycerides. Steps/day fell from the first month to the last month of the trial (by 1870steps/day, *p* < 0.001) and distance deceased (by 1.29km/day).
Bondaronek *et al*., 2022, UK	4/5	Qualitative phenomenological study	25 Healthcare Professionals (GPs, Nurses and Healthcare Assistants)	PA	Qualitative study performed via SSIs exploring barriers and facilitators to eHealth as a concept.	–	COM-B framework used. Themes: Skills gap, training to used tool required Limited tools to support provision of PA advice Time constraints Importance of efficiency and simplicity of tool integration with existing systems. Loss of interpersonal communication
Boudreau *et al*., 2020, Canada	3/5	RCT	242 adults recruited from primary care (IG 131).	PA	3-month intervention assessing web-based toolkit providing 7 15 minute sessions tailored to the individual, exploring education, behavior change and goal setting. The control group received no input.	Ask, Advising, Assisting	Of the 131 in the IG, 80 completed the 3 month follow up. Increased self-reported exercise, with a difference of 58 minutes per week (*p* = 0.04). In IG fall in participation from session 1 (100%) to session 7 (35%) with 61% completion of exit survey. Correlation between number of web-session completed and self-reported exercise levels (*p* < 0.05).
Breeman *et al.*, 2021, Netherlands	3/5	Qualitative phenomenological study	10 patients, 16 healthcare professionals (including nurses, GPs, physiologists) and 1 eHealth company CEO.	PA, diet, alcohol and smoking	Multiple iterative SSIs and FGD with patients, HCPs and business to explore image of stakeholders’ intentions and desire with eHealth.	–	Highlighted 10 core values 1 and 2: continuous care whilst removing burden on HCPs 3 and 4: person centered whilst supporting autonomy of choice. 5: effect at addressing: motivation, adherence and education 6 and 8: inclusion of individuals support and environment, with personal contact with HCP possible 7, 9 and 10: simple, trustworthy and financial viable
Buss *et al*., 2022, Australia	3/5	Qualitative phenomenological study	10 patients with T2 diabetes and CVD recruited from primary care	PA, diet, alcohol and smoking	Development and design of an application as per literature and guidelines, followed by FDGs and likert scale testing in 10 patients.	Ask, Advising, Assisting	Acceptability was tested through 2 FGD (5 per group), with 71% rating the app above average. Feedback from the FGD has led to further app development.
Cai *et al*., 2022, China	4/5	Cluster RCT	72 elderly patients recruited from primary care (IG n = 36)	PA	3 month intervention including face-to-face education, walking groups and app-based peer support versus standard care.	Assisting	64/72 participants completed the study with 2 lost in the IG. At 3 months there was a significant difference in daily steps (as measured by pedometer) between IG and CG (408 steps/day, *p* = 0.03). Also a noted difference in grip strength (1.29kg, *p* = 0.04).
Csaky *et al*., 2021, USA	4/5	Prospective cohort study	40 physical inactive adults recruited from primary care	PA	6 month prospective single group intervention assessing use of activity tracker for assessment of step count for PA promotion.	Ask, Assisting	Physical activity levels increased from baseline by 1,039 steps/day, but not significant, *p* = 0.13. No improvement in self-efficacy for exercise (*p* = 0.877). Intervention was deemed acceptable in survey with ‘more motivated’, ‘inspired’ or ‘accountable’ mentioned.
Dhinagaran *et al*., 2021, Singapore	4/5	Prospective cohort study	60 adults with no significant comorbidities recruited from primary care	PA, nutrition and sleep	4 week intervention delivered by social media addressing lifestyle factors through education. Feasibility study assess usability. No CG.	Advising, Assisting	56/60 (93%) completed the study, with 92% satisfied with the intervention. 54% felt they would recommend to others. 28/56 approved of the intervention acceptability. Median METs-per-week decreased over the intervention (857 v 765). Median mod-vig PA per week increased (30 min v 50 min).
Hawkes *et al*., 2023, UK	4/5	Retrospective cohort study	1826 adults referred to NHS Digital Diabetes Prevention Programme for individuals with type 2 diabetes	PA and nutrition	9 month digital intervention via a mobile application delivering self-monitoring, goal-setting, education and social support. No CG.	Ask, Advising, Assisting.	Outcome measured was application use, not PA levels or health-outcomes. Usage of the app declined from a median of 32 min in month 1 to 0 min in month 9. Self-monitoring of behaviours occurred a median of 117 times. Higher engagement was noted when supported combined with a health coach.
Jang *et al*., 2023, South Korea	4/5	RCT	67 adults with metabolic syndrome (IG 35)	PA	12 week trial assessing affect of telephone feedback on wearable tracker and mobile-app. Controls and IG used tracker and mobile-app, with IG getting 2 weekly phone calls with advise and feedback.	Ask, Advising, Assisting	Non-significant increase in mean steps between the two groups by 2000/day. Improvements noted in metabolic disorder components.
King *et al*., 2020, USA	5/5	Cluster RCT	245 underserved inactive adults in the community (IG 123)	PA	12-month program, with virtual advisor program compared to human advisor program. Participants received 28 brief advising sessions in both IG and CG.	Ask, advising, assisting, and arranging f/u	94.3% of participants completed the 12 month study (95.1% of IG) Mean number of session attended 18.8 in IG compared to 18.4 in control (*p* = 0.76). Results of walking minutes per week supported non-inferiority (153.9 IG v. 131.9 control)
Lemola *et al*., 2021, UK	4/5	Propsectvie cohort study	148 adults in community	PA	3 month app-based prospective cohort study, rewarding outdoor steps with virtual currency to spend in local shops. No CG.	Ask, assisting	70/148 successfully completed the study at 3 months, with 55/148 completing 12 month f/u. Self-reported PA increased from baseline (*M* = −0.12) to 3 months (*M* = 0.22) before returning to baseline at 12 months (*M* = −0.13).
Lugones-Sanchez *et al*., 2022, Spain	5/5	RCT	650 adults recruited from primary care	PA, diet	3 month app-based intervention assessing monitoring via activity tracking with feedback and education. Both IG and CG received standard counselling. Repeat f/u at 12 months	Ask, advising, assisting	563/650 (86.6%) completed 3 month f/u, and 443/650 completed 12 month f/u (68.2%). Median app used was 64.5/90 days. IG showed increase in light activity, vigorous activity and total activity, only light activity increased versus CG at 3 and 12 months.
Mattila *et al*., 2022, Europe	4/5	Secondary analysis of RCT	811 participants recruited from primary care	PA	Secondary analysis of NoHoW trial, an 18 month study assessing a web-based education toolkit. Analysis of participants given access to modules on ‘Goals’ and Barriers’ for PA. Outcomes measured through tracker data.CG received tracker with standard care.	Advising, Assisting	498 (61.4%) visited the Goal module and 406 (50.1%) visited the barriers module. Following the goals module there was no significant change in PA measures, including total active (46.6 v 48.3, *p* = 0.44) Following the barriers module there was only significant change in total activity (45.1 v 46.9, *p* = 0.03), and vigorous activity (24.2 v 24.9, *p* = 0.047)
Mendes *et al*., 2020, Portugal	5/5	Retrospective cohort	Analysis of Portuguese primary care data	PA	Analysis of PA brief assessment tool, counselling tool and app available via PC in Portugal using national PC database.	–	1736/100,000 users of NHS had PA assessed via national platform by variety of PCPs. 94/100,000 Portuguese adults had received PA counselling via eHealth service.
Nau *et al*., 2021, Australia	4/5	Prospective cohort	29 patients from PC with raised BP and 7 GPs	PA, diet, alcohol and smoking	6 months intervention assessing combination of web-based educational content and SMS messaging. Single cohort with SSIs completed at the end to assess acceptability.	Advising, assisting	GPs: time to introduce intervention minimal, receptiveness to intervention limited Patients: 90% read SMS message but limited access of web (53%) and video (52%) over 6 months. High levels of acceptability of intervention, particularly to SMS messaging. No reported impact on behaviors by patients
Parker *et al*., 2022, Australia	5/5	Cluster RCT	215 overweight 40–74 year old in primary care, IG 120.	PA, diet	6 months intervention assessing lifestyle app offering goal setting, monitoring, education and messaging versus standard care. Repeat f/u at 12 month	Ask, Advising, Agreeing, Assisting	Of 120 in IG 85 attended HCP check up, 73 used app and/or coaching with 38 using both. Health literacy at 6 months was improved in IG but not sustained at 12 months. There was also no difference in PA score at 6 or 12 months between IG and CG in ITT analysis.
Pelletier *et al*., 2021, Canada	4/5	RCT	30 adults with T2 diabetes recruited via PC	PA	3 month trial comparing standard PAP with PAP and PA tracking via device. Measuring PA outcome and acceptability.	Ask, Assisting	22/30 completed study with 4 drop-outs in both groups. 86% satisfied or very satisfied with activity tracker use Increase in moderate and vigorous activity in both groups, with no significant difference between groups.
Recio-Rodríguez *et al*., 2022, Spain	4/5	RCT	160 older adults >60 recruited via PC (IG = 81, CG = 79)	PA, diet	3 month trial comparing brief advice, to brief advise with app-based PA and diet tracking with daily personalized feedback.	Ask, Advising, Assisting	IG used application for mean of 70.7/90 days with adherence of 78.5% No difference found in variable attributable to either group. PA (steps/min) −0.4 (−1.0 to 0.2) *p* = 0.174.
Redfern *et al*., 2020, Australia	5/5	RCT	934 patients with or at high risk of CVD recruited via primary care	PA, diet, alcohol and smoking	12 month trial comparing standard advise with a personalized web-site providing assessment, advise, education and messaging that links with EHR.	Ask, Advising, Assisting	451/486 of IG used web-based. Non users (13%, *n* = 58), low users (one log in or more over 3 months of follow up, 47%, *n* = 211) or high users (at least one log in per month, 40%, *n* = 182). Significant increase in PA levels at 12 months, 87% v 79.7%, *p* = 0.02.
Shannahan *et al*., 2021, USA	4/5	RCT	26 patients recruited via primary care, IG 13	PA	12 week intervention of HCP monitoring of activity tracker data. IG and CG both given tracker, with IG received weekly messages from PCP regarding accordance with exercise goals	Ask, Assisting	Of the 13 in in IG, 4 completed the study with 8 lost to follow up and one dropping out due to health reasons. No significant difference between groups in PA or health outcomes. However demonstrated feasibility, with no report of usability.
Stewart *et al*., 2022, USA	4/5	Prospective cohort	33 pre diabetics recruited via PC	PA	6 month intervention of daily SMS messaging with education and weblinks for lifestyle to address pre-diabetes/diabetes. Fitbit used to supplement daily messages. CG received standard care.	Advising Assisting	Significant increased noted in days of moderate PA/week (difference 2.0, *p* = 0.015), days of vigorous PA/week (difference 1.5, *p* = 0.035) and total PA (difference 62.4mins/wee, *p* = 0.039). Usability not tested.
Taylor *et al*., 2020, UK	5/5	Mixed-methods RCT	450 adults with CVD risk factors. IG numbers 224	PA	12 month intervention comparing standard Exercise Referral Scheme (ERS) with ERS plus eCoaching to build behavioral skills, assessed by pedometer based PA tracking.	Advising Assisting	109/224 in IG met the accelerometer wear time criteria, compared to 128/226 in CG. Indicative but non significant improvement in mod-vig PA at 12 months, 11.8mins/week difference (*p*−0.10)
Wattanapisit *et al*., 2021, Thailand	5/5	Qualitative phenomenological study	16 PCPs	PA	FGD with PCP exploring the feasibility and challenges of an mHealth application in PC.	–	4 themes developed: 1. Application for personalized PA counselling 2. Barriers: technical difficulties and integration 3. Patient involvement, not tech savvy, personal device needed 4. Impact on services: time consuming, requiring technical support.
Wattanapisit *et al*., 2021, Thailand	5/5	Qualitative phenomenological study	15 PCPs (3 doctors, 12 nurses) from PC	PA	Phenological qualitative FGD and SSIs of PCPs to explore perspective on the development of an eHealth tool to aid PA counselling.	–	Three themes emerged 1. Evidence based and tailored 2. Easy to use, prescription function, tracking and recalls as able 3. Low time consuming, use in busy clinic, deals with patients limitations
Woldamanuel *et al*., 2023, Sweden	5/5	Qualitative phenomenological study	14 patients with T2 diabetes or pre-diabetes and 10 PCPs	PA	Phenological qualitative FGD of patients -and SSIs of PCPs to explore perspective on the development of an eHealth tool to aid PA counselling.	–	Three themes emerged 1. Utility (motivation, cohesion platform, support) 2. Adoption (personalized, adaptable, not suitable for everyone) 3. Accountability (digital skills support, confidentiality, liability)
Young *et al*., 2020, USA	4/5	RCT	319 adults with diabetes (IG *n* = 158), recruited from PC	PA	3 month intervention assessing standard care versus health coaching, mHealth and PA tracking with data integrated into patient health record, with f/u at 9 months.	Ask, Assisting	High retention rate of 89.9% in IG IG steps significantly increased from 23700 at baseline to 39167 at 3 months, and 32601 at 9 months.

### Analysis

Descriptive statistics were used to present quantitative data. Each intervention was categorised according to the stage of action using the *5A’s model* and highlighted in the WHO BRIEF project (*integrated brief interventions for noncommunicable disease risk factors in primary care: the manual*) (Anderson *et al*., 2022). The model provides an evidence-based structure for healthcare professionals to address health promotion. The stages of the 5A’s model are: (1) ask and measure exposure to risk factors (2) advise patients to change exposure to risk factors, (3) assess readiness to change, (4) assist patients in acquiring the motivation, self-help skills or support needed and (5) arrange follow-up support (Anderson *et al*., 2002; Glasgow *et al*., 2006).

The effectiveness of a digital health intervention was determined based on whether the study reported statistically significant positive outcomes in comparison to a control, normal care or baseline condition. Acceptability was considered only when explicitly assessed and reported by the study authors, such as through user satisfaction, engagement metrics or qualitative feedback. All assessments of effectiveness and acceptability were based solely on self-reported data as presented in the original studies, with no additional secondary analysis conducted.

Qualitative data were analysed using a conventional content analysis, as described by Hsieh and colleagues (Hsieh and Shannon, 2005), and this was used to identify patterns within qualitative data to allow for systematic coding and categorisation. The results were obtained via the following steps: (a) familiarisation with the papers by one author; (b) identification of previously identified themes from qualitative papers were re-coded into sub-categories; (c) these sub-categories were then categorised into themes according to similarities and differences. All themes were initially developed by one author and finalised in discussion with other authors.

## Results

### Summary of search results and study selection

**Figure 1.**
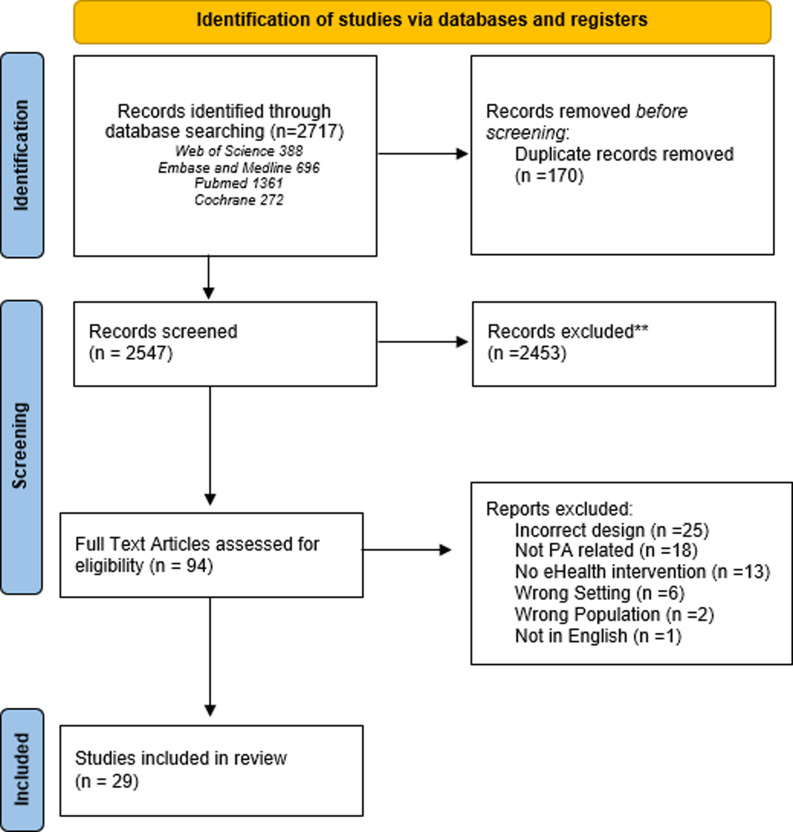
PRISMA flow diagram.

Our systematic search retrieved 2,717 studies, including 170 duplicates, leaving a total of 2,547 studies for review. Following title and abstract screening, 94 studies were included for full text review. We identified 29 studies for inclusion, as shown in the PRISMA flow diagram (Figure 1).

Of the included studies, 13 were randomised control trial, 2 mixed-method studies, 6 prospective cohort studies, 2 retrospective cohort studies and 6 qualitative phenological studies. Study quality was mixed, with nine deemed high, seventeen moderate and three low quality as assessed by the MMAT scoring system.

### Lifestyle counselling domains

**Figure 2.**
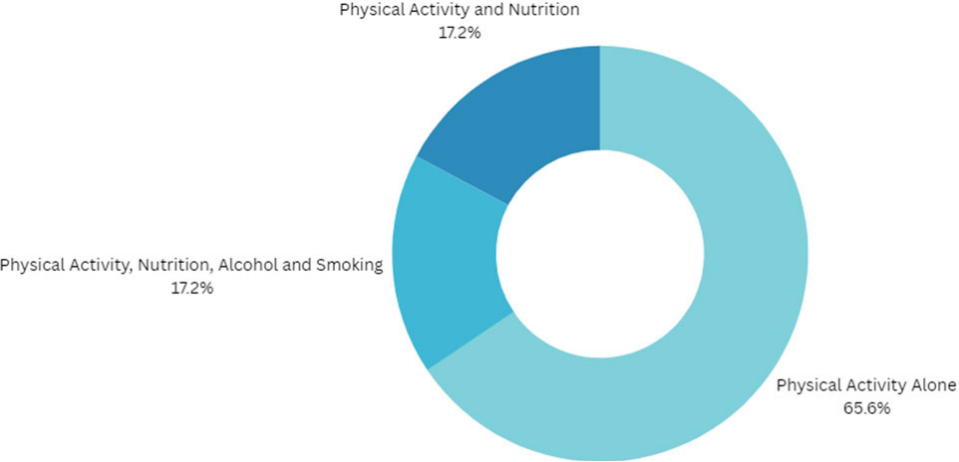
Digital Health interventions separated by counselling domains they deliver (*n* = 29).

Of the twenty-nine studies included in this scoping review, nineteen used interventions focused solely on PA. Five studies focused on interventions that integrated the four other domains of lifestyle medicine: PA, nutrition, alcohol and smoking. The final five studies focused on interventions combining PA and nutrition (Figure 2).

### Method of digital health intervention delivery

**Figure 3.**
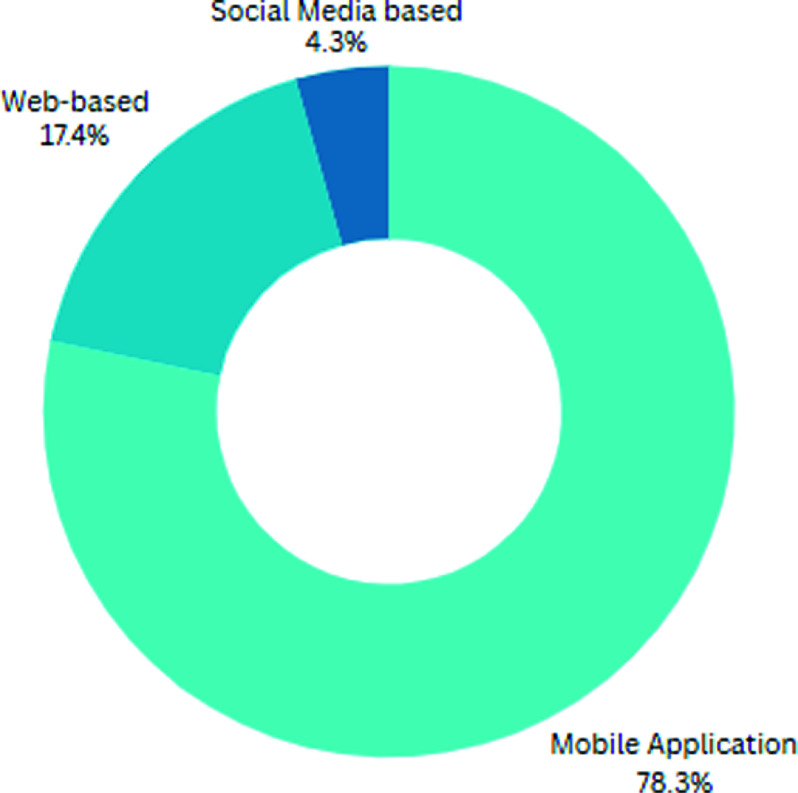
Method of delivery of digital health intervention (*n* = 23).

Twenty-three out the twenty-nine studies (79%) evaluated or assessed a specific digital health intervention. Of these studies, eighteen (78%) investigated a mobile application, with twelve of these combined with a fitness tracker. A further four (17%) digital health interventions were web-based, with one intervention using social media messaging to deliver education and motivation (Figure 3).

### Method of implementation

Of the 23 studies that evaluated a specific digital health intervention, each was categorised based on how it aligned with the 5A’s model for behavioural counselling (Ask, Advise, Assess, Assist, Arrange), as summarised in Table 3. Most interventions addressed multiple components of the model, though with varying emphasis.

**Table 3.** Method of implementation of PA promotion

Area of intervention of eHealth tool as described by 5A’s model	eHealth model count (same model may be counted multiple times for different areas) (total number = 3)
Ask	16
Advise	14
Assess	1
Assist	22
Arrange	1

The **“**Ask**”** component—assessing PA levels—was addressed in 16 of the 23 studies (70%), typically through self-reported questionnaires. The **“**Advise**”** step, involving personalised recommendations to increase PA, was identified in 14 studies (61%). Only one study explicitly incorporated the **“**Assess**”** step, which involves evaluating an individual’s readiness or confidence to change behaviour.

The **“**Assist**”** category was the most commonly addressed, with 22 interventions (96%) providing tools or strategies to support behaviour change. This included features such as PA tracking (*n* = 12, 52%) and educational content (*n* = 15, 65%) aimed at enhancing motivation and self-efficacy. Finally, the **“**Arrange**”** step—typically referring to planning follow-up or referrals—was present in only one study (4%).

### Effectiveness and acceptability

Nineteen studies reported on the effectiveness of the digital health intervention on increasing PA. Of these, eight did not report any significant improvements in PA due to the digital health intervention when compared to control groups. However, eleven studies (57%) did report significant improvements in PA levels of digital health intervention users. No studies identified that the digital health interventions decreased PA levels of patients when compared to standard care or no intervention.

**Figure 4.**
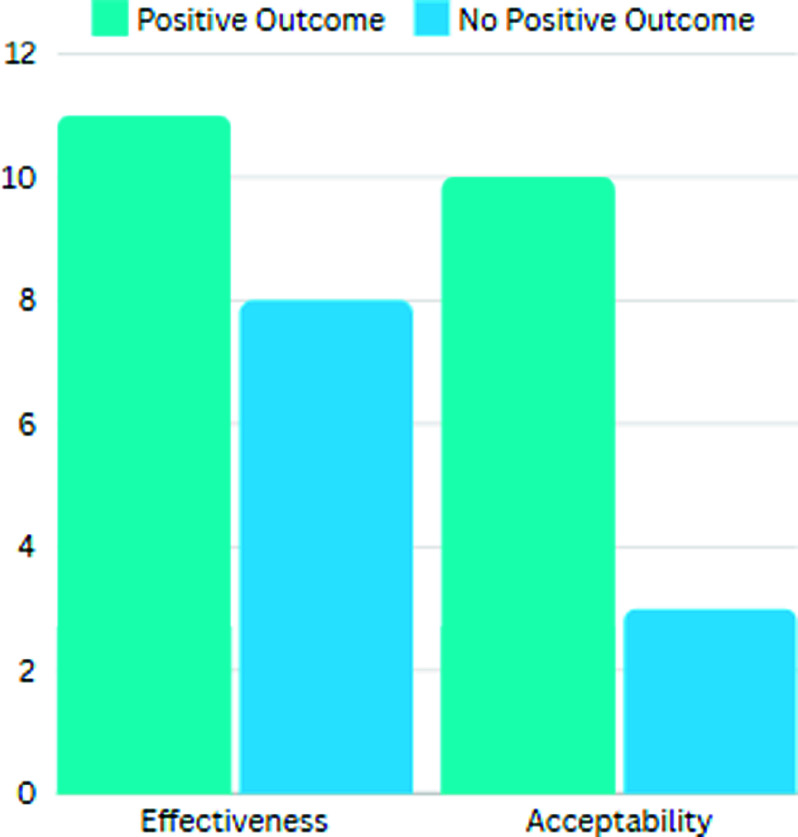
Acceptability and effectiveness of digital health interventions, presented by number.

Of the studies that reported on the acceptability of the digital health intervention, ten presented positive outcomes (see Figure 4). Four of these studies reported a high completion rate in the intervention group, implying a translation of acceptability into practice (Dhinagaran *et al*., 2021; King *et al*., 2020; Lugones-Sanchez *et al*., 2022; Young *et al*., 2020).

Three studies showed poor uptake on the digital health intervention (Hawkes *et al*., 2023; Mattila *et al*., 2022; Parker *et al*., 2022). All of these were education or advisory based without an exercise-tracking capacity, with one(Hawkes *et al*., 2023) finding engagement was higher when support was combined with a health-coach.

### Content analysis

Six of the studies included in this scoping review (Bondaronek *et al*., 2022; Breeman *et al*., 2021; Buss *et al*., 2022; Wattanapisit, *et al*., 2021; Wattanapisit, *et al*., 2021; Woldamanuel *et al*., 2023) were qualitative phenomenological studies, which explored stakeholder (patients, primary healthcare practitioners and tech businesses) views of digital health interventions. From these papers three main themes emerged:

1. Ease of use. This was important for both practitioners and patients, and simplicity was identified as an essential component (Bondaronek *et al*., 2022; Breeman *et al*., 2021; Wattanapisit, *et al*., 2021; Wattanapisit, *et al*., 2021; Woldamanuel *et al*., 2023).
2. The need for any digital health intervention to act as an adjunct to primary healthcare. Healthcare professionals are time pressured, and therefore interventions need to be easy to implement with minimal time investment and a strong evidence-base regarding effectiveness. As an extension of this, and in-keeping with the need for simplicity, a desire was expressed for any digital health intervention to require minimal resourcing. Finally, to deliver a holistic and combined service, a need for integration with pre-existing systems and electronic health records is required (Bondaronek *et al*., 2022; Breeman *et al*., 2021; Wattanapisit, *et al*., 2021).
3. Digital health interventions should be patient centred. All stakeholder groups expressed a wish for personalised interventions that support the individual and their autonomy, whilst also offering the possibility of interpersonal communication if required (Bondaronek *et al*., 2022; Breeman *et al*., 2021; Wattanapisit, *et al*., 2021; Wattanapisit, *et al*., 2021; Woldamanuel *et al*., 2023).

## Discussion

### Comparison with existing literature

This scoping review identifies the wide variety (related to both method and means) of digital health interventions. Although the majority of included studies (65.5%) focussed solely on PA, other lifestyle domains were included in some digital health interventions, including nutrition, alcohol and smoking cessation advice. The integration of different lifestyle domains is likely to impact outcome, as outlined in a recent narrative review (Leese *et al*., 2024). An uplift might be particularly pronounced when PA and nutrition counselling are co-delivered (Johns *et al*., 2014). However, outcomes appear to be impaired when smoking cessation advice is co-delivered with other lifestyle interventions(Meader *et al*., 2017; Schulz *et al*., 2014).

In this scoping review, 78% of all digital health interventions were delivered by a mobile application. A plethora of PA mobile applications exist, with over 150,000 existing in 2017 (Kennedy and Hales, 2018). There is a lack of standardisation between mobile applications, with research highlighting they are frequently limited in their scope, function or compliance with the WHO PA guidelines (Foster, 2019; Schoeppe *et al*., 2017). This lack of standardisation, makes assessment of effectiveness and validity challenging (Baker *et al*., 2010).

The categorisation of digital health interventions into a recognised behaviour change model (5A’s) has, to the best of the author’s knowledge, not previously been done. By providing a framework for analysis it allows the identification of what features contribute to digital health intervention effectiveness and acceptability.

The results in our study regarding the acceptability of digital health interventions for PA promotion in primary healthcare combined with previous research (Gonçalves *et al*., 2022) support the stance of the WHO in their *Global Action Plan on PA (WHO, 2019)*, which highlights technology interventions as a viable and strategic means of engaging patients in PA and supporting health-related behaviour change. The results from the content analysis provides clear guidance as to what patients, practitioners and digital health-developers desire: a patient-centred application which provides autonomy, is simple to use and works as an adjunct to ongoing primary healthcare services. To act as an adjunct for primary healthcare, it must be quick and simple to use and integrate with pre-existing systems.

Despite the endorsement by the WHO regarding digital health interventions being acceptable, this review found no conclusive evidence as to their effectiveness. There are several possible reasons for this, including: (1) different abilities and needs of distinct patient populations, (2) changing intergenerational needs and desires, (3) technological literacy and availability existing along chronological and geographical disparities and (4) healthcare-system differences. Alongside this variability in studies, the absence of clear evidence is exacerbated due to a poor quality of existing studies (Eland-de Kok *et al*., 2011; Zangger *et al*., 2023).

A key consideration for the broader implementation of digital health interventions is how they complement and enhance the work of primary healthcare providers. While many of the reviewed studies focused on patient-facing outcomes, no studies included in this review explored whether digital health interventions increased the rate of PA promotion by primary care professionals or addressed the communication pathways between digital tools and healthcare professionals. Although Mendes and colleagues (Mendes *et al*., 2020) explored the utilisation of digital health interventions in Portuguese primary healthcare, it is not clear whether this represents any change to pre-intervention levels of PA promotion. For a digital health intervention to be effective within a primary care, it is likely that primary healthcare professionals need to be engaged with the intervention and informed of patient progress and outcomes. This could occur through integration with electronic health records, automated alerts or summary reports that support clinical decision-making.

### Strengths and limitations

This scoping review followed the previously published protocol and the PRISMA guidelines outlined for reporting scoping reviews (Tricco *et al*., 2018). In a rapidly changing context, particularly in light of the recent COVID-19 pandemic, this provides an up-to-date overview of digital health interventions and to the best of our knowledge this is also the first study to have defined the method of digital health intervention by a well-known behaviour change model (5 A’s model). Only including papers from January 2020 until December 2023 (as per protocol) was decided in the context of previous work (Wattanapisit *et al*., 2020).

There are several limitations. Given defined inclusion criteria, the exclusion of any non-English studies, grey literature and abstracts may have resulted in a loss of some data. The scope of the search terms used was relatively narrow, a pragmatic and realistic response to time and resource constraints. While the review highlights a range of functionalities that digital health interventions can offer—including supporting, motivating, monitoring and promoting PA—these broader concepts were not fully reflected in the initial search strategy. As a result, it is possible that some relevant interventions were not captured. Future research should incorporate a wider range of search terms to better reflect the full spectrum of digital health interventions used to promote PA. The data-extraction and thematic analysis was conducted by a single author as part of a pragmatic decision-making process, however the effect of this was minimised by a 10% check for reliability by a second author. Given the aims of a scoping review to provide a holistic overview of the literature, although a study quality appraisal was performed, no studies were subsequently excluded. Furthermore, to allow for the inclusion of a wide variety of studies, a meta-analysis was not performed. A future meta-analysis would allow for quantification of the effectiveness of digital health interventions to assist the promotion of PA in primary healthcare.

### Implications for practice

Of all the studies included, none looked at the impact of a digital health intervention on practitioners’ attitudes and behaviours. With the knowledge that PA promotion is effective at increasing patients levels of PA (Haskell, 2003) and is cost-saving (Campbell *et al*., 2015), future research should explore the impact of technologies on practitioners attitudes and behaviours towards PA promotion.

Given the need to increase PA levels across all populations, covering the spectrum of age, health and geography, more robust large scale randomised-control trials with long -term follow-up across all these groups are required. The needs of different groups may not align (for example adolescent versus elderly) and so the inclusion of qualitative work to assess acceptability in these groups is also of value.

This review also sought to categorise the methods of action of the digital health interventions seeking to deliver PA promotion according to a recognised behaviour change model- the 5A’s framework. The results show the majority of interventions (96%) focused on “assisting” individuals to be more active. Interventions incorporating components that “assess” readiness to change and “arrange” on-going follow-up were far less common. This highlights a gap in the integration of tailored behavioural assessment and long-term structured follow-up in digital health interventions to support PA promotion, which could be vital for sustaining behaviour change over time. Future research should explore the role of including components that incorporate behavioural assessment and follow-up in digital health interventions.

The future of healthcare involves the integration of technology, and this includes PA promotion (WHO, 2019). The availability of technology is this field is already vast (Kennedy and Hales, 2018), but evidence-based and trustworthy tools need to be created. The content analysis offers a useful oversight of stakeholder wishes and can guide future work to standardised the approach, for example by a delphi-study (Baker *et al*., 2010). This has the potential to enhance the evidence base and lead to quicker implementation in national policy and on a global scale.

## Conclusions

This scoping review found that digital health interventions for PA promotion in primary healthcare were acceptable, but findings regarding effectiveness were mixed. Findings from included qualitative studies provide clear guidance as to what stakeholders’ desire from digital health interventions in this area, with future output likely to benefit from clarity on application development and means of evaluation. Finally, further work is needed to evaluate the impact of digital health measures on practitioners’ PA attitudes and behaviours as well as patient PA levels.
